# A 2D Photographic and 3D Digital Dental Model Analysis of Golden Percentage in Maxillary Anterior Teeth

**DOI:** 10.1155/2021/6674400

**Published:** 2021-04-17

**Authors:** Naseer Ahmed, Mohamad Syahrizal Bin Halim, Zuryati Ab Ghani, Zafar Ali Khan, Maria Shakoor Abbasi, Nafij Bin Jamayet, Mohammad Khursheed Alam

**Affiliations:** ^1^Prosthodontics Unit, School of Dental Sciences, Health Campus, Universiti Sains Malaysia, 16150 Kubang Kerian, Kota Bharu, Kelantan, Malaysia; ^2^Department of Prosthodontics, Altamash Institute of Dental Medicine, Karachi 75500, Pakistan; ^3^Conservative Dentistry Unit, School of Dental Sciences, Health Campus, Universiti Sains Malaysia, 16150 Kubang Kerian, Kota Bharu, Kelantan, Malaysia; ^4^Hospital Universiti Sains Malaysia, 16150 Kubang Kerian, Kota Bharu, Kelantan, Malaysia; ^5^Department of Oral & Maxillofacial Surgery and Diagnostic Sciences, College of Dentistry, Jouf University, Sakaka, Al Jouf 72345, Saudi Arabia; ^6^Division of Clinical Dentistry, Prosthodontics, School of Dentistry, International Medical University, Jalan Jalil Perkasa 19, Bukit Jalil, Kuala Lumpur 57000, Malaysia; ^7^Department of Preventive Dentistry, College of Dentistry, Jouf University, Sakaka, Al Jouf 72345, Saudi Arabia

## Abstract

The objective of this paper was to evaluate the existence of golden percentage in natural maxillary anterior teeth with the aid of 3D digital dental models and 2D photographs. And to propose regional values of golden percentage for restoration of maxillary anterior teeth. For this purpose, one hundred and ninety dentate subjects with sound maxillary anterior teeth were selected. Standardized frontal images were captured with DSLR, and the apparent width of maxillary anterior teeth was measured utilizing a software on a personal laptop computer. Once the dimensions were recorded, the calculations were made according to the golden percentage theory (GPT). The data were analyzed by independent and paired *T*-test. The level of significance was set at *p* < 0.05. The golden percentage values were not found in this study. The values obtained were 16%, 15%, 20%, 20%, 15%, and 16% moving from the right canine to the left canine teeth. There was no significant gender difference in the golden percentage values. Thus, golden percentage should not be used solely for the correction of anterior teeth or for determining dental attractiveness. Emphasis should be given to a range of dental proportion on regional basis.

## 1. Introduction

In the exploration to create esthetically agreeable restoration of lost and traumatic maxillary anterior teeth, researchers have proposed the application of aesthetic proportions to define the correlation between the widths of maxillary anterior teeth [1]. Lombardi was the first to recommend the use of the harmonious proportion in dentistry. He suggested that it is essential to develop a balance between the dentition and the facial profile. Therefore, he recommended the Golden Proportion Theory (GPT) for the creation of an esthetic anterior tooth dimensions, which is a constant ratio of 1.618 : 1 [2]. He concluded that the widths of the maxillary anterior teeth are repeated in this proportion. In addition, it was also suggested by Levin [3] that, when the maxillary anterior teeth are evaluated from frontal plane, the width of the lateral incisor should be in golden proportion to the width of central incisor and width of canine to the lateral incisor. A grid with columns in golden proportion was invented by him and later on proposed, to use the grid for evaluation and development of harmonious maxillary anterior teeth restorations [3]. However, the recent literature reported that variations have been observed in dental features; hence, in aesthetically pleasing smiles, the golden ratio was not found [4–7].

As an alternate, other principles were introduced for the implementation of esthetic restoration in the labial segment including RED (recurring esthetic dental proportion), (Preston Proportion) and GP (golden percentage). The RED was recommended by Ward [8, 9]. He described it as “the proportion of the successive width of the teeth remaining constant when progressing distally from the midline.” Moreover, Preston studied the occurrence of golden proportion and found that it only existed in 17% of the subjects [10]. Therefore, he suggested Preston's proportion, which states that “the width of maxillary lateral incisor should be 66% the width of central incisors and the width of maxillary canines should be 55% the width of maxillary central incisors when observed from the frontal view.” Consequently, Snow proposed a bilateral ratio for each anterior tooth in percentage of the total apparent intercanine width. He named it golden percentage theory (GPT), as it was a modification of the golden proportion concept. GPT states that the width of maxillary central incisor should be 25%, lateral incisors 15%, and canines should be 10%, respectively, of the intercanine distance, as measured from the distal surface of one canine to the other [11]. This concept has been investigated by researchers and suggested ethnic variations in percentage around the globe [12–14]. This study evaluated the existence of golden percentage in a selected sample of Pakistani population. The purpose of this study was to evaluate the presence of GPT between natural maxillary anterior teeth in local adult population. The evidence from the current study could be used as reference for the restoration of the esthetically demanding anterior segment and may be used to reestablish the dimensions of teeth during periodontal surgery, restorative dentistry, and prosthetic rehabilitation clinically.

## 2. Materials and Methods

### 2.1. Study Setting and Sample Size

This study was carried out at Altamash Institute of Dental Medicine Karachi (AIDM), Pakistan. The sample size was calculated with public service of creative research systems survey software (creative research systems, version 9, Petaluma, California, United States). Considering 62% [13] prevalence of dental proportion and estimated sample size at 5% margin of error with 95% confidence interval, 227 individuals with intact natural maxillary anterior teeth were invited to participate in this study, considering the 10,000,000 population.

### 2.2. Ethical Consideration and Participant Recruitment

The ethical and review board of AIDM (AIDM/EC/06/2019/06) and Universiti Sains Malaysia (USM/JEPeM/19060380) approved this study. The objective, consent statement for voluntary participations and declarations of anonymity and confidentiality were clarified and sought out for all participants before participation in this study. Students, employees, coworkers, and other staff studying/working at the campus were interviewed first and examined clinically; those who fitted our inclusion and exclusion criteria were selected. 37 participants were excluded based on selection criteria, and a total of 190 subjects were included in the study. The age and nationality of participants was noted and confirmed by national identity card. The basic information, form number, age, gender, and contact details were recorded.

The inclusion criteria were individuals with intact dentition, caries and periodontal disease-free anterior teeth, lack of faciodental asymmetry, and no restorative treatment done in anterior teeth, i.e., veneers, crown, and bridge work. Patient's age ranges from 18 to 30 years on the date of examination.

### 2.3. 2D Photograph and 3D Digital Model Making

A digital camera (Canon EOS, DSLR Camera, CMOS, 18 MP,1920 X 1080p/30fps) body was used in this study. The camera was equipped with a built-in magnification lens of 18 − 55 mm + 75 − 300 mm to capture crisp clear and reproducible images. The 1 : 1 macrosetting was used for close-up imaging of teeth and generally included the four maxillary incisors and canine on the sensor. The camera was mounted on a tripod, set at 12 o'clock position with a standardized focus and place at a distance of 1.5 meters from the subjects to ensure distortion free images. The surrounding lighting remained the same for all the photographs. Ring flashlight source system (LED-FD,480II, Medike Photo and Video Co., Ltd. Yidoblo, Guangdong, China) was used, and its configuration consisted of a flash unit that were mounted next to the lens. The height of the lens of camera was adjusted on the tripod to match the incisors level for retracted smile image capture. Subjects were seated upright with shoulders and head held straight and facing forward, towards the lens of the camera. Head position was standardized along both horizontal and vertical axes. Then, the upper lip was retracted in all intraoral photographs to clearly display the maxillary anterior teeth. This procedure was similar to the protocol described by Bidra et al. [15] as presented in Figure S3.

For 3D measurement of maxillary arch, impressions were made of all subjects with irreversible hydrocolloid impression material on their maxillary arch (fast setting alginate hydrogum; Zharmack Spa) and were poured with type IV dental stone (ISO Type 3, Elite Rock Zharmack Spa). The casts were coded with serial number of the subjects using permanent marker. In order to obtain a 3D model, the cast was scanned by UP3D Dental Laboratory Scanner (UP360+, 300 × 300 × 400 mm, 3D scanner, Shenzhen, China). The scanner was equipped with 2.0 MP cameras that can scan with high precision upto 6 *μ*m. The full arch 3D scan was obtained in 20 seconds. The scan image was displayed on a compatible dental design software (UPCAD, UP3D, Shenzhen, China), then transferred via USB to store in a personal computer as described in Figure S2.

### 2.4. Validity and Reliability Control

In our study, all data from 2D photographs and 3D dental models were collected by a single operator. To overcome interoperator biasness, 20% of the photographs and models were initially measured by a senior working colleague. The readings obtained were compared with calculations carried out by the principal investigator. The correlation obtained between the operators reading was 0.79 in current study. Furthermore, to minimize intraoperator biasness, firstly, each measurement was performed three times, and the mean value was calculated. Secondly, 20% of models and photographs were remeasured by the same operator after a period of two weeks. A strong correlation of 0.89 was found after analysis through Dahlberg's formula ME = (*Σ* (*x*1 − *x*2)2/2(28))1/2.

For validity purpose, 20% of data from direct tooth measurement via a sharp tip digital vernier caliper was compared with actual tooth widths obtained through digital dental models and assessed through intraclass correlation coefficient test (ICC). A strong correlation value of 0.81 was found.

To avoid magnification error by figuring a conversion factor which was derived by dividing dimensions of cast teeth by the image dimension [7], the width of each tooth was multiplied by the factor in order to find out the true width captured in the photographs.

### 2.5. Teeth Measurement

The actual width of the anterior teeth was recorded with 3D scanner software on the models. Mesiodistal widths (actual width) of central incisors, lateral incisors, and canines were measured from the facial side using measuring tool device positioned between the contact points of each tooth from contact to contact point of teeth as presented in Figure S1(a).

Then, the obtained images were processed in Photoshop 2020 (Adobe, version 21.0.2, San Jose, California, United States). The width of teeth between contact points in central incisors and visible width of lateral incisor and canine teeth on each side of the arch were measured from labial aspect as shown in Figure S1(b). The data collected was recorded in separate computer sheets for future analysis.

### 2.6. Golden Percentage Calculation

**Figure 1:**
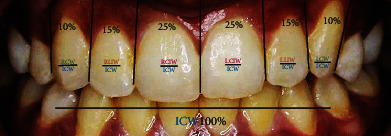
The methodological assessment of golden percentage between the widths of maxillary anterior teeth in this study. RCIW: right central incisor width; RLIW: right lateral incisor width; RCW: right canine width; ICW: intercanine width; LCIW: left central incisor width; LLIW: left lateral incisor width; LCW: left canine width.

For estimation of golden percentage of maxillary anterior teeth widths, the mesiodistal distance of central, lateral incisors and canine teeth from each side of the arch was divided by the intercanine width and further multiply by 100 to obtain percentage values. If the values obtained were 25%,15%, and 10% from right to left canine teeth, it was considered that the teeth are in golden percentage (Figure 1).

### 2.7. Statistical Analysis

The data was analyzed with Statistical Package for the Social Sciences Software (IBM, SPSS statistics, version 25, Chicago, Illinois, United States). Descriptive analysis of qualitative and quantitative variable, i.e., age, gender and golden percentage, was carried out to calculate their mean, standard deviation, and percentage values. Furthermore, mean values of dependent (anterior teeth width, golden percentage) and independent (age, gender) variables were compared using independent and paired *T*-test. The distribution of data was analyzed with normality plots and testing (Shapiro-Wilk and Kolmogorov-Smirnov). A *p* value ≤ 0.05 was taken as statistically significant.

## 3. Results

This descriptive analytical study consisted of 190 participants. The response rate was 83.70% and dropout rate 16.29%, respectively. In our study, 79 (41.6%) participants were male and 111 (58.4%) females with a mean age of 21.9 ± 2.62 years. Normal distribution curve was observed in data of most independent variables, i.e., maxillary anterior teeth width and dental proportion values.

The mean width for upper right central incisor (URCI) was 7.99 ± 0.61, 6.07 ± 0.82 for upper right lateral incisor (URCI) and 6.68 ± 1.11 for upper right canine (URC), while 7.87 ± 0.79 for upper left central incisor (ULCI), 6.01 ± 0.83 upper left lateral incisor (ULCI), and 6.42 ± 1.18 for upper left canine (ULC) teeth, respectively. The intercanine width was 41.07 ± 3.85 amongst the participants. There was a significant difference between mean width of right and left central incisors, canine teeth (*p* ≤ 0.001) and (*p* ≤ 0.001) while no significant difference (*p* = 0.293) was seen between the mean width of lateral incisor teeth on both sides of the arch as described in Table 1.

**Figure 2:**
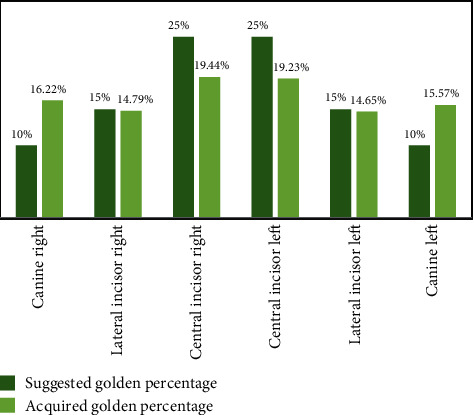
The difference between the golden percentage suggested by Snow and dental proportion obtained in this study (*n* = 190).

Furthermore, the mean golden percentage values for anterior teeth were 19.44% for upper right central incisor (URCI), 14.79% for upper right lateral incisor (URLI), 16.22% for upper right canine (URC), 19.23% for upper left central incisor (ULCI), 14.65% for upper left lateral incisor (ULCI), and 15.57% for upper left canine (ULC), respectively, as depicted in Table 2. There was a significant difference (*p* < 0.05) between the values obtained in this study and the one proposed by Snow 25%, 15%, and 10% as mentioned in Figure 2.

The mean dental proportion width in respect to gender obtained in our study is presented in Table 3. The mean values were (starting from the right canine side to left upper canine teeth) 16.22%, 14.79%, 19.44%, 19.23%, 14.65%, and 15.57%. Moreover, there was no significant difference between dental proportion of teeth amongst male and females (*p* > 0.05) in all anterior teeth except right lateral incisor (*p* = 0.011) and (*p* = 0.014). The relationship between values of dental proportions obtained in this study and golden percentage theory suggested by Snow [11] is described in Figure 2. Additionally, there was a significant difference (*p* = 0.026) between the mean teeth widths obtained from 3D models and 2D photographs in this study.

However, no statistically significant difference was found between the width of the right (*p* = 0.505) and left (*p* = 0.741) central incisors, similarly between right (*p* = 0.780) and left canines (*p* = 0.645) in females. Although a statistically significant difference was seen (*p* = 0.030) between right lateral incisor widths in females. Additionally, there was no statistically significant difference between the widths of the right (*p* = 0.510) and left central incisors (*p* = 0.742), also in right (*p* = 0.776) and left canines (*p* = 0.647) amongst male participants. Whereas a statistically significant difference was found amongst the widths of right lateral incisors (*p* = 0.031). Lastly, there was no significant difference between the mean intercanine width in male (*p* = 0.602) and female (*p* = 0.605) as mentioned in Table 4.

## 4. Discussion

The formation of a geometric relationship in anterior teeth of maxilla is essential in order to make an esthetically acceptable restoration. It would be convenient and beneficial if statistically reliable relationships are created, to support teeth proportion theories [16]. In this regard, most analysis is carried out on photographs and dental casts widely; however, attempts have been made by several researchers to minimize the photographic magnification error [7, 16, 17]. In this study, W.H Ward concept was used to avoid magnification error by figuring a conversion factor, which was derived by dividing dimensions of cast teeth by the image dimension [7].

The dental proportion values in our study were in contrast with standard golden percentage [11] as we found a value of 16.22%, 14.79%, 19.44%, 19.23%, 14.65%, and 15.57%, from the right canine to left canine teeth. Murthy and Ramani found a dimension of 12.5%, 15.5%, 22%, 22%, 15.5%, and 12.5% in front teeth [14] on standardized teeth images from 56 dental students of Asian origin. Maharjan and Joshi in their research on Mongoloid/Aryan population found a golden percentage of 22.19-22.48% in central incisors, 15.95-15.47% in lateral incisors, and 11.30-11.91% for canine teeth in female, whereas 11.26%, 15.52%, 22.61%, 22.56%, 15.65%, and 12.37% in males, respectively [17]. The dimensions of teeth from these studies along with the present study differ from values of standard golden percentage, proposed by Snow, who recommended a value of 25% for central incisors, 15% for lateral incisors, and 10% for canines. The variations in the values may be attributed to the racial differences [11]. Likewise, numerous other studies have found similar results and culminate no relation between perceived tooth dimension and proposed values of the golden percentage recommended by Snow [11]. However, an exception is seen with the lateral incisors, which was around 15% in manifold studies, although the width of central incisors was lesser and the width of canines was greater than dimensions of teeth proposed by Snow [11, 18–21]. Therefore, it was proposed that GPT would be befitting if the values are modified by taking into consideration the ethnicity of population.

Furthermore, the mean GPT values of central incisors in our study had a range of 19.3-19.54% as far as females are concerned, while for males, it was 19.0-19.54%. These values are smaller than those recommended by Snow [11]. Conversely, the mean values for lateral incisors ranged from 14.5 to 14.6% for females and 14.6-15.1% for males were in accordance with the proposed value of 15% for lateral incisors [11]. Similar values for lateral incisors were found in numerous studies [12, 18–21]. According to our study, the mean dental proportion value was 15.7-16.2% in females and 15.3-16.2% in males. These figures are 10% greater than the one proposed by Snow [11]. Therefore, it was found that the widths of both central incisor and canine teeth were either slightly smaller or larger than the standard values of GPT [11]. These findings were in accordance with Ali Fayyad [12] and other similar studies [18–21]. Hence, we recommend a value of 20% for centrals, 15% for laterals, and 16% for canines to be implemented as the values relevant to natural dentition, especially to Pakistani population.

Although, it is not appropriate to standardize all patients in need for rehabilitation of the anterior teeth in the same way as every person, dental practitioners need some fundamental guidelines during the treatment planning phase [22, 23]. As the esthetics varies significantly from one individual to another, it is essential to consider the dentofacial specificities of each individual and the wide variety of natural teeth proportions when restoring or replacing the maxillary anterior teeth.

The present study had some limitations like conventional impressions recorded and then digitized using 3D scanner software. Intraoral scanners have been practiced in dentistry, thus allowing a completely digital workflow, from impression to final restoration, with clinical reliability and satisfactory patients' feedback [24]. In future studies, digital impressions could be recorded with an intraoral scanner that can be easily linkable with an image software. Furthermore, digital impression is more efficient, convenient, and faster than the conventional impression [25].

However, the sample size selected for the current study was comparable with previous studies, but the data collection was confined to a single center only, as the differences in racial characteristics might influence the result of an esthetic dentistry-related study, especially in a population which has wide cultural diversity. Therefore, in future, a multicenter study with a large sample size and cultural diversity is recommended.

## 5. Conclusions

In luminosity of the fallouts of this study, it is concluded that the standard golden percentage values were not pertinent on the participants of current study; therefore, golden percentage should not be used solely for the correction of anterior teeth or for determining dental attractiveness. Therefore, emphasis should be given to a range of mathematical values, on regional basis.

## Figures and Tables

**Table 1:** Characteristics of maxillary anterior teeth widths (*n* = 190).

Variables	Range	Minimum	Maximum	Mean	Std. deviation	*p* value
RCIW	3.80	6.20	10.00	7.99	0.612	0.001
LCIW	4.40	5.30	9.70	7.87	0.796	
RLIW	4.10	4.20	8.30	6.07	0.820	0.293
LLIW	5.30	4.10	9.40	6.01	0.838	
LCW	6.00	3.10	9.10	6.42	1.189	0.001
RCW	5.90	3.00	8.90	6.68	1.118	

RCIW: right central incisor width; RLIW: right lateral incisor width; RCW: right canine width; ICW: intercanine width; LCIW: left central incisor width; LLIW: left lateral incisor width; LCW: left canine width.

**Table 2:** Distribution of golden percentage in maxillary anterior teeth (*n* = 190).

Variables	Minimum	Maximum	Mean	Std. deviation
GpRCI	10	24	19.44	0.02
GpRLI	12	20	14.79	0.01
GpRC	9	21	16.22	0.02
GpLCI	15	26	19.23	0.01
GpLLI	12	21	14.65	0.01
GpLC	8	19	15.57	0.02

GpRCI: golden percentage right central incisor; GpRLI: golden percentage left lateral incisor; GpRC: golden percentage right canine; GpLCI: golden percentage left central incisor; GpLLI: golden percentage left lateral incisor, golden percentage left canine.

**Table 3:** Characteristics of golden percentage relation between maxillary anterior teeth in both gender (*n* = 190).

Variables	Gender	*n*	Mean	S.td	Minimum	Maximum	*p* value
Golden percentage Central incisor right side	Male	79	19.54	0.01	17.0	23.0	0.990
	Female	111	19.54	0.01	17.0	24.0	0.989
Golden percentage Lateral incisor right side	Male	79	15.11	0.01	12.0	20.0	0.011
	Female	111	14.55	0.01	12.0	18.0	0.014
Golden percentage Canine right side	Male	79	16.2	0.01	11.0	21.0	0.943
	Female	111	16.2	0.02	9.0	21.0	0.941
Golden percentage Central incisor left side	Male	79	19.0	0.014	16.0	23.0	0.297
	Female	111	19.3	0.018	15.0	26.0	0.278
Golden percentage Lateral incisor left side	Male	79	14.6	0.015	12.0	21.0	0.975
	Female	111	14.6	0.014	12.0	20.0	0.976
Golden percentage Canine left side	Male	79	15.3	0.019	8.0	18.0	0.296
	Female	111	15.7	0.021	8.0	19.0	0.291

**Table 4:** Distribution of mean maxillary teeth width in respect to gender (*n* = 190).

Variable	Gender	Mean	*t* value	*p* value
RCIW	Female	7.967	0.667	0.505
	Male	8.027	0.661	0.510
RLIW	Female	5.963	2.182	0.030
	Male	6.224	2.179	0.031
RCW	Female	6.664	0.280	0.780
	Male	6.710	0.285	0.776
LCIW	Female	7.894	0.331	0.741
	Male	7.855	0.330	0.742
LLIW	Female	5.998	0.398	0.691
	Male	6.047	0.388	0.699
LCW	Female	6.463	0.461	0.645
	Male	6.382	0.459	0.647
ICW	Female	40.950	0.518	0.602
	Male	41.247	0.523	0.605

RCIW: right central incisor width; RLIW: right lateral incisor width; RCW: right canine width; ICW: intercanine width; LCIW: left central incisor width; LLIW: left lateral incisor width; LCW: left canine width; *t* value indicates the degree of variation or difference in sample data; ICW: intercanine width.

## Data Availability

The raw data used to support the findings of this study are included within the article.

## Supplementary Materials

Supplementary Materials Figure S1: (a) 3D dental cast analysis; (b) 2D photograph analysis. Figure S2: 3D digital dental cast workflow diagram. Figure S3: pictorial illustration showing methodology of obtaining standard digital images with subject in natural head position.
