# Supplementary material for: A 2D Photographic and 3D Digital Dental Model Analysis of Golden Percentage in Maxillary Anterior Teeth
Source: Biomed Res Int. 2021 Apr 17;2021:6674400. doi: 10.1155/2021/6674400 (PMC8081617; doi:10.1155/2021/6674400)
Supplement: Supplementary Materials — Figure S1: (a) 3D dental cast analysis; (b) 2D photograph analysis. Figure S2: 3D digital dental cast workflow diagram. Figure S3: pictorial illustration showing methodology of obtaining standard digital images with subject in natural head position. [file 6674400.f1.docx]

**Supplementary Material**


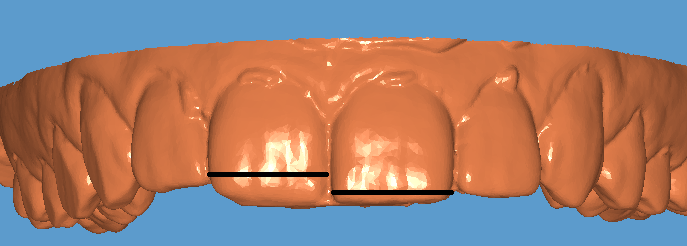


A


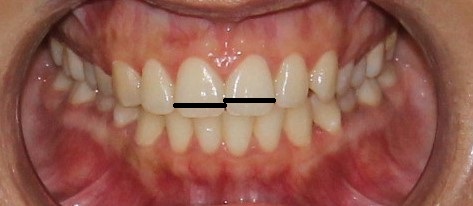


B

**Figure S1: (A); 3D dental cast analysis, (B); 2D photograph analysis**


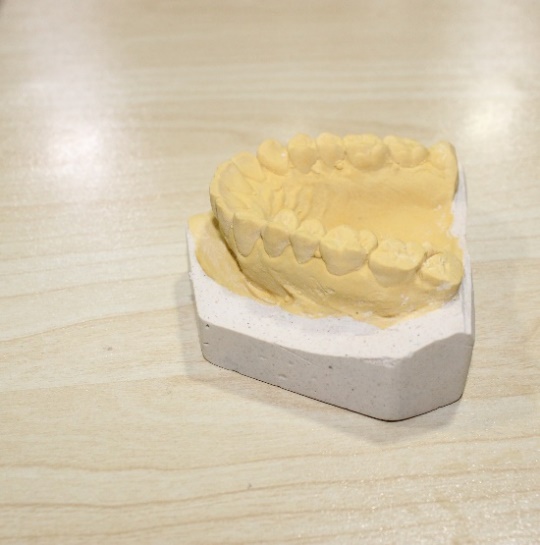

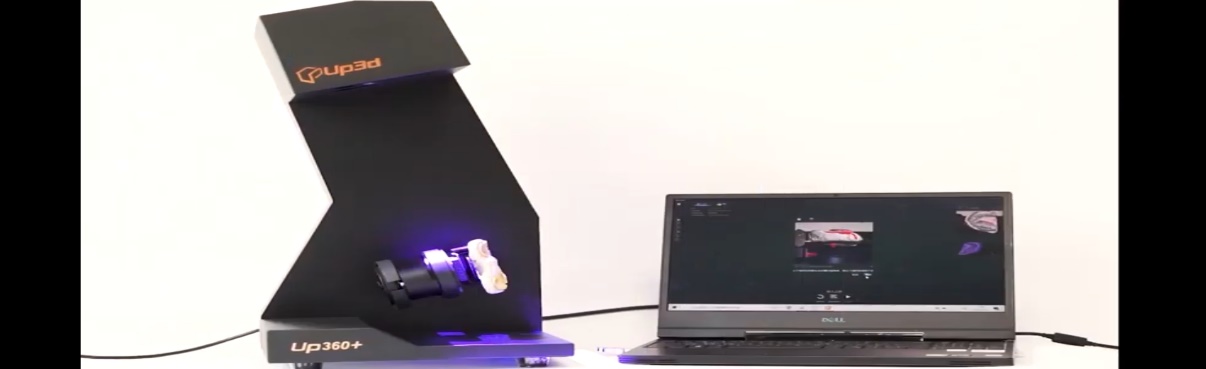

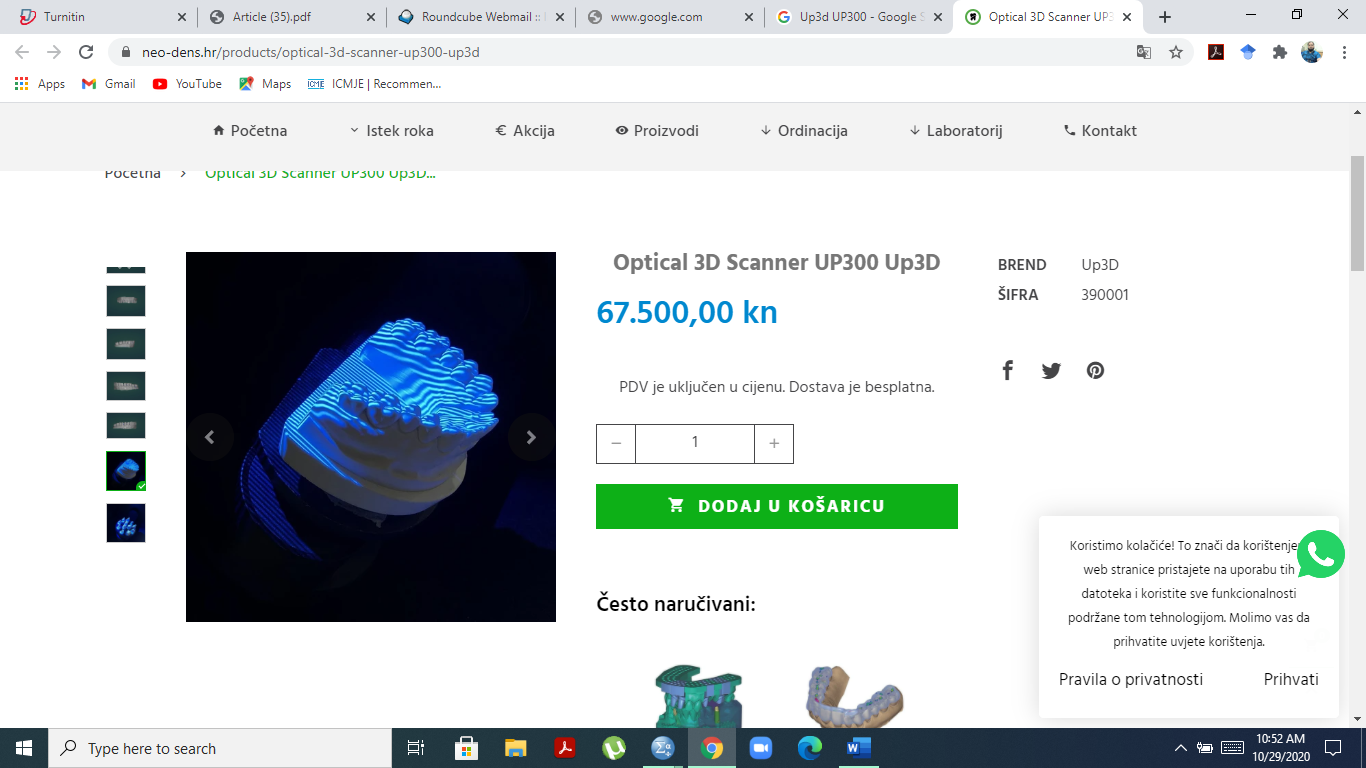


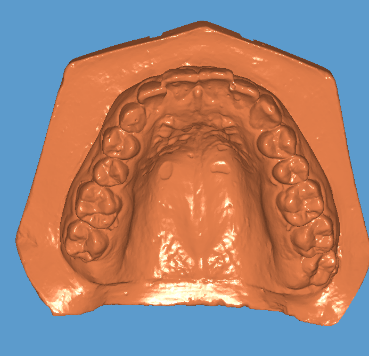


**Figure S2:** A 3-D digital dental cast work flow diagram


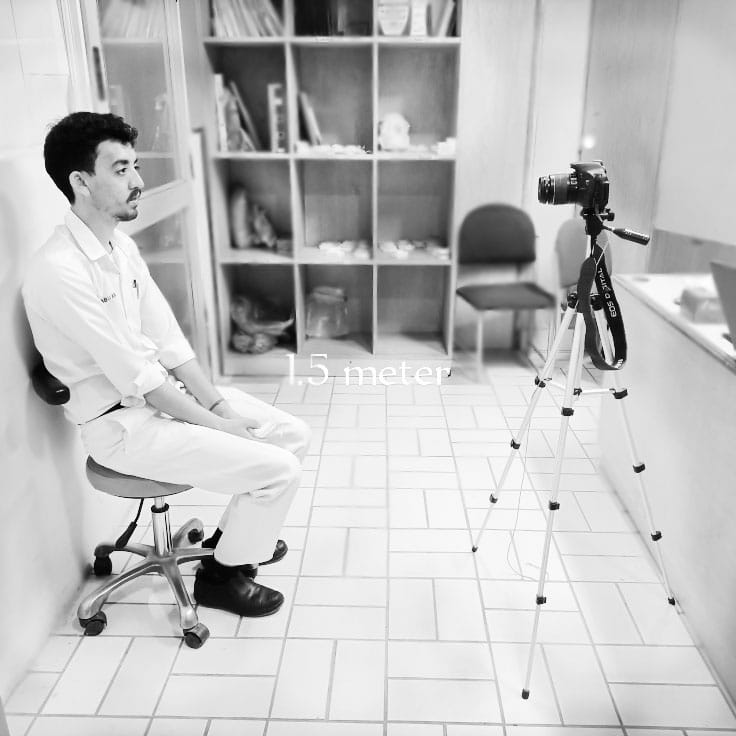


**Figure S3:** Pictorial illustration showing methodology of obtaining standard digital images with subject in natural head position. The distance from camera to subject was 1.5 meter.
